# Diverse Functions of Tim50, a Component of the Mitochondrial Inner Membrane Protein Translocase

**DOI:** 10.3390/ijms22157779

**Published:** 2021-07-21

**Authors:** Minu Chaudhuri, Anuj Tripathi, Fidel Soto Gonzalez

**Affiliations:** Department of Microbiology, Immunology, and Physiology, Meharry Medical College, Nashville, TN 37208, USA; atripathi@mmc.edu (A.T.); Fgonzalez18@email.mmc.edu (F.S.G.)

**Keywords:** Tim50, TOM, TIM, Trypanosoma, TIMM50, HAD-phosphatase family

## Abstract

Mitochondria are essential in eukaryotes. Besides producing 80% of total cellular ATP, mitochondria are involved in various cellular functions such as apoptosis, inflammation, innate immunity, stress tolerance, and Ca^2+^ homeostasis. Mitochondria are also the site for many critical metabolic pathways and are integrated into the signaling network to maintain cellular homeostasis under stress. Mitochondria require hundreds of proteins to perform all these functions. Since the mitochondrial genome only encodes a handful of proteins, most mitochondrial proteins are imported from the cytosol via receptor/translocase complexes on the mitochondrial outer and inner membranes known as TOMs and TIMs. Many of the subunits of these protein complexes are essential for cell survival in model yeast and other unicellular eukaryotes. Defects in the mitochondrial import machineries are also associated with various metabolic, developmental, and neurodegenerative disorders in multicellular organisms. In addition to their canonical functions, these protein translocases also help maintain mitochondrial structure and dynamics, lipid metabolism, and stress response. This review focuses on the role of Tim50, the receptor component of one of the TIM complexes, in different cellular functions, with an emphasis on the Tim50 homologue in parasitic protozoan *Trypanosoma brucei*.

## 1. Introduction

**Figure 1:**
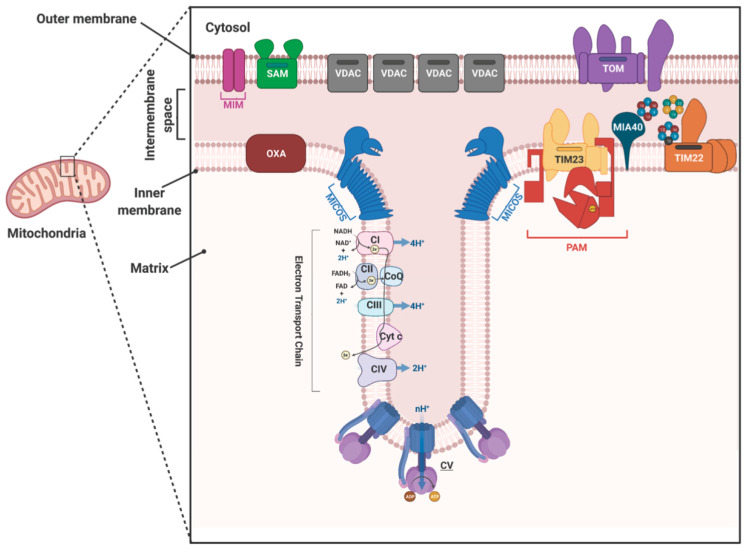
Schematics of protein complexes of the mitochondrial outer and inner membranes as identified in yeast. Mitochondrial cristae junction and the contact site for the outer and inner membranes are enlarged. The Sorting and Assembly of the β-barrel proteins (SAM), MIM, and the translocase of the outer membrane (TOM), are involved in the import of β-barrel, α-helical, and all other nuclear-encoded mitochondrial proteins in and through the mitochondrial outer membrane, respectively. The voltage-dependent anion channel (VDAC) mediates small molecules and metabolite fluxes through the mitochondrial outer membrane. TIM23 and TIM22 are the major protein translocases of the mitochondrial inner membrane. Presequence translocase (TIM23)-associated motor complex (PAM) imports protein into the matrix. Small Tim complexes acts as chaperones for hydrophobic inner membrane proteins to cross the intermembrane space. MIA40 and OXA1 imports proteins to the intermembrane space and the inner membrane, respectively. Mitochondrial contact site and cristae organization system (MICOS) form the cristae junction. Respiratory complexes and ATP synthase (CI, CII, CIII, CIV, and CV) are localized in the cristae membrane. The picture was created with the BioRender.com.

Mitochondria are evolved from the endosymbiont proteobacteria [1]. Thus, like its ancestor, mitochondria are surrounded by double membranes. The mitochondrial outer membrane (MOM) is semipermeable due to the presence of multiple pores created by the most abundant mitochondrial protein, voltage-dependent anion channel (VDAC) that allows fluxes of small molecules and metabolites [2]. Whereas mitochondrial inner membrane (MIM) is impermeable even to ions and is folded inwards to form cristae [3]. MIM separates two aqueous compartments in the mitochondria, the innermost matrix and inter membrane space (IMS). At the invagination site, MIM forms a tubular structure, known as cristae junction site, that is held together near the periphery by a large protein complex known as the mitochondrial contact site and cristae organizing system (MICOS) [4]. Mitochondrial respiratory complexes and ATP synthase are localized in the cristae membrane and are functionally coupled via chemiosmotic proton pump that is required for energy production [5] (Figure 1).

The size of mitochondrial proteomes varies among eukaryotes; ~1800 in mammals, ~2000 in plants, and ~1000 in yeast [6,7,8]. The residual genome of the bacterial endosymbiont only encodes 1% of these proteins and the rest of the proteins (99%) are nuclear DNA encoded [8,9,10]. These proteins are synthesized by the cytosolic ribosomes and stay unfolded by binding through cytosolic chaperones till they are recognized by the receptor translocases of the TOM [8,9,10]. Most of the mitochondrial proteins are in the MIM and the matrix thus require crossing both membranes [8]. The MOM and IMS also possess several important proteins [11,12]. Proteins are translocated through TOM and TIMs either as fully or partially unfolded conditions [8,9,10]. Once reached their destination, newly translocated proteins are folded properly by chaperones for being functional. Translocation of proteins through TIM requires mitochondrial membrane potential. In addition, ATP hydrolysis is needed for proteins to enter mitochondrial matrix. The contact site between the MOM and MIM facilitates protein import by bringing TOM near one of the TIM complexes [13]. To be discriminated from the other cytosolic proteins, mitochondrial proteins harbor at least one mitochondrial targeting signal (MTS). In most of the cases MTS is located at the N-terminal; however, certain proteins have multiple internal targeting signals. The MTSs do not have any consensus sequence, although N-terminal MTSs possess certain characteristics. It consists of an amphipathic α-helix with several positively charged residues positioned on one side of the helix and the hydrophobic residues face the other side of the helix [14]. The internal MTSs are less characterized. Tim50, a component of the one of the two TIM complexes found in fungi, plants, and animals recognizes the N-terminal MTS-containing proteins for translocating them through the MIM [8,9,10].

## 2. Mitochondrial Protein Translocases

Mitochondrial protein import is mostly studied for the eukaryotic supergroup opisthokonta, that includes fungi and mammals [8,9,10,15]. There are parallel studies from plants [16] and most recent advances are made from other eukaryotic supergroups including excavatae that includes many pathogenic protists [17,18,19,20]. Previous studies from fungi, mammals, and plants revealed that at least eight protein complexes are required to traffic proteins to different locations in mitochondria (Figure 1); (1) TOM complex imports most of the mitochondrial proteins [21], (2) the sorting and assembly (SAM) complex in the MOM imports and assembles MOM β-barrel proteins [22], (3) MOM protein import (MIM) complex imports other MOM proteins with α-helical TMDs [23], (4) mitochondrial IMS assembly (MIA) complex is required for import and folding of several IMS-proteins [12], (5) TIM23 and (6) TIM22 are two major translocases of the MIM that imports proteins to the matrix, MIM and IMS [24,25], (7) presequence translocase associated motor (PAM) complex mediates translocation of preproteins to the matrix at expense of ATP hydrolysis [26], and (8) cytochrome oxidase assembly (OXA) complex inserts/imports some MIM proteins [27] (Table 1). Most of these translocases are multi-protein complexes (Table 1). The subunits are overall conserved among fungal, mammals, and plants translocases; however, some variations are present. The most divergent protein import machineries are found so far in a parasitic protozoan *Trypanosoma brucei* (Table 1) [17,18].

**Figure 2:**
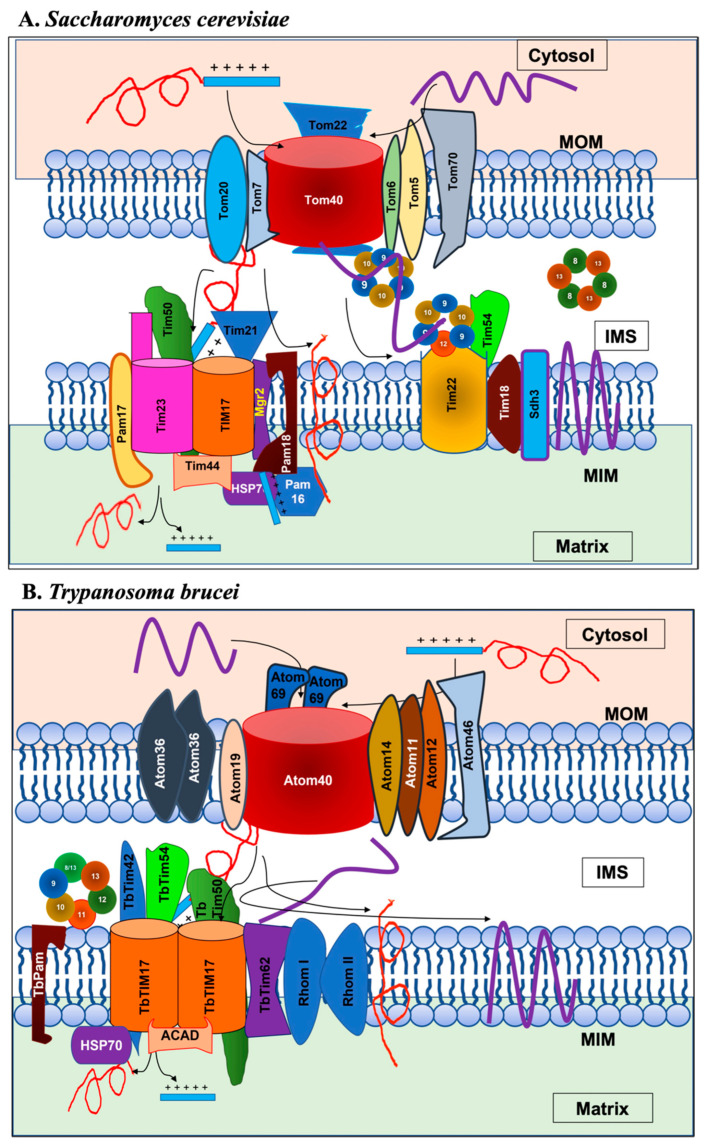
(**A**) Translocases of the mitochondrial outer and inner membranes, TOMs and TIMs, in *Saccharomyces cerevisiae*. The TOM complex consists of Tom40, Tom70, Tom20, Tom22, Tom5, Tom6, and Tom7. The two TIM complexes are TIM23 and TIM22. The core components of the TIM23 complex are Tim23, Tim17, and Tim50. Tim21 helps connect TIM23 with TOM and with respiratory complex III (not shown). Mgr2 couples Tim21 to the TIM23 core. TIM23 associates with the PAM complex consisting of Tim44, Hsp70, Pam16, Pam17, Pam18, and MgeI. The major component of the TIM22 complex is Tim22, and other components include Tim54, Tim12, Tim18, and Sdh3. The five small Tims are Tim8, Tim9, Tim10, Tim12, and Tim13. Tim9 and Tim10 as well as Tim8 and Tim13 form two separate heterohexameic complexes in the IMS to carry cargo proteins from the TOM complex to the TIM22 complex. (**B**) The ATOM complex and the single TIM complex in *Trypanosoma brucei*. The major component of the ATOM complex, Atom40, and other subunits Atom14, Atom11, Atom12, Atom19, Atom46, and Atom69 are shown. Atom36 often associates with Atom40 but is not a part of this complex. The major component of the TbTIM complex is TbTim17. TbTim62, TbTim42, Rhom I, and Rhom II are integral membrane proteins. TbTim50 is also membrane-integrated but possesses an IMS-exposed C-terminal domain. TbTim54 is a peripherally associated IMS protein. The location of ACAD is not known but expected to be in the matrix. Recent studies have shown TbPam27 as membrane bound. Small TbTims are associated with TbTim17. The cytosol, mitochondrial outer membrane (MOM), intermembrane space (IMS), mitochondrial inner membrane (MIM), and matrix are labeled.

The translocation pathway of the nuclear-encoded mitochondrial proteins depends on their destination. Here, we will focus only the translocation of proteins through the TOM and TIM complexes. To enter the mitochondrial matrix, a protein generally needs an N-terminal mitochondrial targeting signal (MTS), which the receptor of the TOM complex Tom20 recognizes (Figure 2A). Then, Tom22, an integral membrane component of the TOM complex, recognizes the MTS. Consequently, the protein enters the TOM channel formed by Tom40, a major component of the TOM complex [28,29]. Once the protein emerges in the inter membrane space (IMS) from the Tom40 channel, the MTS associates with the trans site of Tom22, allowing the TIM23 complex to recognize it [30,31]. The MIM has two TIM complexes: TIM23 and TIM22 as named according to the pore forming units of these complexes, Tim23 and Tim22, respectively (Figure 1 and Figure 2A) [32,33]. Like TOM, both TIM23 and TIM22 are multi-protein complexes. The core components of TIM23 are Tim23, Tim17, and Tim50. Tim23 and Tim17 each have four transmembrane domains (TMDs) in the center, with their N- and C-termini exposed to the IMS. Tim17 associates with Tim23 and acts as the structural component of the TIM23 channel [34]. Tim50 has a single TMD, with the N-terminus facing the matrix and the larger C-terminus facing the IMS. The C-terminus acts as the receptor for MTSs that emerge from the TOM channel [35,36]. The mitochondrial membrane potential is essential for proteins to cross the MIM. Matrix-targeted proteins require the presequence translocase-associated motor (PAM) complex to enter the matrix [37]. The PAM complex consists of membrane peripheral component Tim44 that recruits Hsp70. The other PAM components include J-domain containing protein Pam18, J-like protein Pam16, co-chaperone Mge, and yeast-specific protein Pam17 (Figure 2A) [9,10]. ATP hydrolysis by Hsp70 provides the energy and direction for the active translocation of preproteins. Once the preproteins are in the matrix, the mitochondrial processing peptidase (MPP) cleaves the presequence or MTS, and the Hsp60/Hsp10 chaperone complex folds the resulting proteins [8,9,10].

TIM23 also imports some MIM proteins that possess a sorting signal consisting of a stretch of hydrophobic residues, along with the N-terminal targeting signal. After crossing the mitochondrial outer membrane (MOM) through the TOM channel, these proteins are recognized by the TIM23 complex [8,9,10]. Once the sorting signal reaches the import channel, translocation stops. Then, Tim21, a single TMD-containing protein, displaces Pam18. Next, the TIM23 channel opens laterally and the translocating protein is inserted into the MIM [38,39]. On the other hand, the TIM22 complex imports MIM proteins that do not contain the N-terminal MTS but instead possess multiple TMDs (Figure 2A) [40,41]. These are primarily mitochondrial carrier proteins and translocase components Tim17, Tim23, and Tim22. The heterohexameric complex formed by Tim9 and Tim10 or Tim8 and Tim13 (for translocation of Tim23) in the IMS chaperone these hydrophobic proteins to the TIM22 channel [42,43]. Animals and plants also have similar TOM and TIM complexes; however, the individual components vary among different species, particularly those of the TIM22 complex (Table 1).

*Trypanosoma brucei* is a unicellular protozoan that causes African trypanosomiasis, a fatal disease in human and domestic animals [44]. The disease is transmitted by the bite of the Tsetse fly that is prevalent in sub-Saharan Africa, thus confining the disease primarily to the African continent. Treatment options for this disease are limited since most available drugs are antiquated and toxic as well as promote resistance [45]. While recent efforts from the World Health Organization (WHO) have significantly reduced the disease burden, political unrest and economic downfall often undermine disease surveillance efforts. As a result, some 36 million people are at a constant risk of contracting this disease [https://www.who.int/news-room/fact-sheets/detail/trypanosomiasis-human-african-(sleeping-sickness) (accessed on 12 July 2021)].

*T. brucei* belongs to the eukaryotic supergroup excavata that also includes other parasitic protozoa like *T. cruzi* and *Leishmania*. Many of these parasites cause devastating diseases in humans worldwide [46]. This group of eukaryotes diverged very early during evolution, thus possessing many unique machineries for performing basic cellular functions [47,48,49]. Trypanosomatids possess a single tubular mitochondrion per cell. Their mitochondrial genome consists of many circular DNAs concatenated to form a disc-like structure known as kinetoplast [50]. Despite its complexity, the mitochondrial genome in these parasites only encodes 12–18 proteins. Therefore, like in other eukaryotes, most of the mitochondrial proteins in these parasites are encoded in the nuclear genome and imported into the mitochondrion after synthesis by cytosolic ribosomes [17,18]. Proteomic analysis has shown a *T. brucei* mitochondrion to require about 900 different proteins for its function [51,52]. A uniquely divergent protein translocation machinery in *T. brucei* imports and sorts these proteins into different sub-mitochondrial locations [17,18].

*T. brucei* possesses a TOM complex known as ATOM [53,54]. The major component of ATOM is Atom40, an archaic homologue of Tom40. Other proteins in ATOM include Atom11, Atom12, Atom14, Atom19, Atom46, and Atom69 [53,54] (Figure 2B). *T. brucei* also possesses a TIM complex [55,56]. The major component of the *T. brucei* (Tb) TIM complex is TbTim17 [55,56]. Unlike other eukaryotes which possess three homologous proteins, Tim17, Tim23, and Tim22, *T. brucei* only possesses TbTim17 [57]. TbTim17 exists in large protein complexes with sizes ranging from 300 to 1100 kDa by associating with other TbTims [55,56]. These include TbTim62, TbTim42, TbTim50, TbTim54, two Rhomboid-like proteins, acetyl CoA dehydrogenase (ACAD), and six different small TbTims (Figure 2B) [55,56,58,59,60]. TbTim17 and TbTim62 each possess four predicted TMDs, whereas Tim42 and Tim50 have a single TMD near the C- and N-terminal region, respectively. Rhomboid-like proteins have multiple TMDs [56,58]. The six small TbTims are TbTim9, TbTim10, TbTim11, TbTim12, TbTim13, and TbTim8/13 [61,62]. Except TbTim12, all small TbTims have a pair of characteristic CX3C motifs that resemble those in small Tims in other eukaryotes. On the other hand, TbTim12 only has one C in each of these motifs [61]. Human and yeast small Tims form a pair of intra-molecular disulfide bonds and have a hairpin-like structure [63,64]. Structural modeling has shown that all small TbTims can form a similar hairpin structure. Small TbTims do not have any TMD; these proteins are tightly associated with TbTim17 [61,62]. Harsman and colleagues showed that most of these TbTims are likely involved in the import of both the N-terminal and internal signal-containing mitochondrial proteins, suggesting that *T. brucei* has a single TIM complex [56]. It is also probable that different accessory components are associated with the core module of the TbTIM17 complex to import different substrate proteins. TbTim17 and TbTim50 are the most conserved components found in *T. brucei*. This review focuses on the structure and function of Tim50 in different eukaryotes, with an emphasis on TbTim50.

## 3. Discovery of Tim50

Three groups independently discovered Tim50 to be an essential component of the TIM23 complex in *S. cerevisiae* and *N. crassa*. Geissler et al. replaced the endogenous copy of Tim23 with a 2X-protein A-tagged Tim23 and purified the protein complex from yeast mitochondrial extract using IgG-Sepharose. Besides the known components Tim17, Tim23, and Hsp70, they also detected a new 50-kDa protein and showed it to be essential for translocating matrix-targeted preproteins [65]. Yamamoto et al. arrested a fusion protein containing the N-terminus (220 AAs) of cytochrome b2 (pCytb-220) and dihydrofolate reductase (DHFR) during its translocation into yeast mitochondria by adding methotrexate to the reaction mixture. In doing so, they photo-crosslinked a new protein with this substrate [66]. Purification of the cross-linked product and mass spectrometry analysis identified Tim50. Mokranjac et al. identified the Tim50 homologue in *N. crassa* while purifying its TIM23 complex [67]. They inactivated the endogenous copy of Tim23 by repeat-induced point mutation (sheltered RIP) while expressing an ectopic copy of N-terminal His-9-tagged Tim23. Solubilization of the mitochondrial proteins with digitonin-containing buffer and purification of the complex by Ni-NTA resin followed by anion-exchange chromatography identified a 56-kDa protein along with Tim23 and Tim17, two major components of the TIM23 complex [67]. Further characterization of this 56-kDA protein confirmed it as the homologue of Tim50. Drosophila Tim50 was later identified in studies on mutant flies defective in growth and development [68]. Using P-insertional mutagenesis, Moritoh et al. identified an X-linked semi-lethal line GP99 with slower growth and poor larval development. Molecular cloning identified the mutant gene as the homologue of *Tim50* and the authors named it *tiny tim50* (*ttm50*). In mammals Tim50 is referred as TIMM50. Guo at al. characterized the human TIMM50 homologue [69]. While attempting to identify proteins that interact with TRAIL-receptors DR4 and DR5 by yeast-two-hybrid, the authors identified a 40-kDa protein. Further characterization revealed this protein to be the homologue of yeast Tim50. This protein localizes to the mitochondria and the endogenous protein does not interact with DR5, although its C-terminal domain can interact with the death domain of DR5 in vitro. Guo et al. showed that unlike yeast and fungal Tim50, human TIMM50 has a dual-specific phosphatase activity [69]. In the same study, the authors also showed that Tim50 depletion in zebrafish caused defects in developmental regulation that resulted in neurodegeneration, dysmorphic hearts, and reduced motility due to increased cell death. It has been suggested that these defects were likely due to loss of mitochondrial membrane potential in Tim50-depleted cells [68,69]. Different plant species also possess homologues of Tim50 as a part of their Tim17-Tim23 complex; however, functional characterization of these homologues is limited. Our laboratory identified a homologue of Tim50 in *T. brucei* [58]. Syntenic sequences of Tim50 homologues are also present in the genomes of other kinetoplastid parasites like *T. cruzi* and different species of *Leishmania* [58]. Like human TIMM50, TbTim50 possesses a dual-specific phosphatase activity [58]. Overall, Tim50 homologues exist in almost all eukaryotes, except for *Giardia* and a few amoebic protozoa.

## 4. Tim50 Primary Structure and Membrane Topology

**Figure 3:**
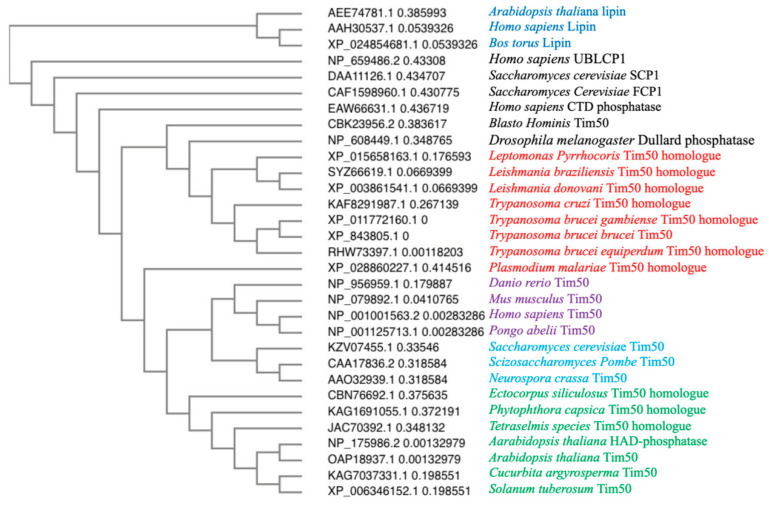
Phylogenetic analysis of the Tim50 homologues and few HAD-phosphatase family proteins from different organisms. Sequences were aligned in PHYLIP format and guided tree was generated using CLUSTAL Omega server. The image shows a Neighbour-joining tree without distance correction. Accession number for each protein in the NCBI protein database is indicated. Tim50s in plants (green), yeast/fungi (blue), animals (purple), and trypanosomatids (red) are indicated by different colors as indicated. Other HAD phosphatases and Lipins are shown in black and darker blue, respectively.

**Figure 4:**
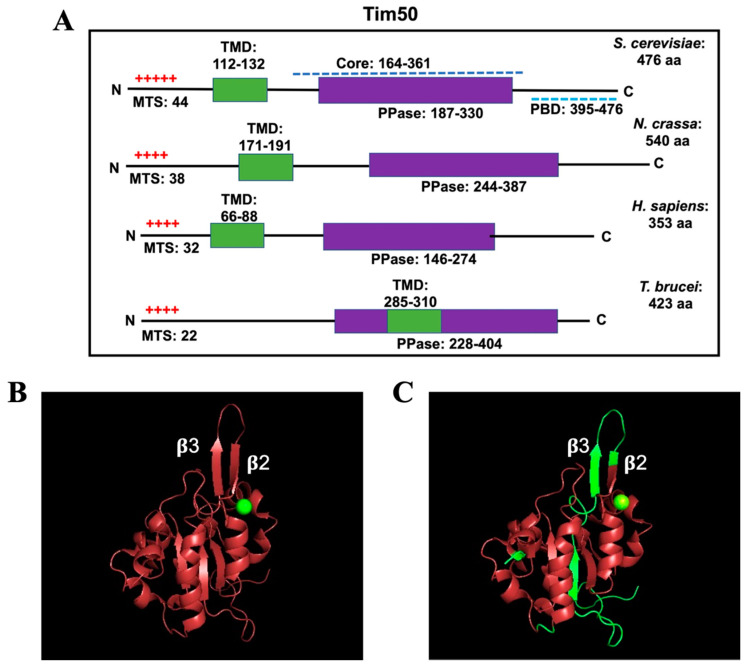
(**A**) Schematics of Tim50 proteins from *S. cerevisiae*, *Neurospora crassa*, *Homo sapiens, and T. brucei*. The length of the N-terminal mitochondrial targeting signal (MTS) for each protein is indicated. The number of red plus signs indicates the number of charged residues within each MTS. The transmembrane domain (TMD) is shown in green, and the CTD phosphatase-like domain is shown in purple. The amino acid (AA) residues are numbered. The core and presequence-binding domain (PBD) in ScTim50 are shown. (**B**,**C**) Structural homology modeling using the Cn3D program. The crystal structure of the ScTim50_IMS_ core region (PDB ID 4QQF) was used as a template for comparing the predicted structure of hTim50 (**B**) and TbTim50 (**C**).

Tim50 homologues are present in most eukaryotes (Figure 3). According to the NCBI protein database, the length of Tim50 ranges from 320 to 576 amino acids (AAs). However, some of the shorter Tim50 proteins have not been fully characterized. Well-characterized Tim50 proteins include *N. crassa* (Nc) Tim50 (540 AAs), *S. cerevisiae* (Sc) Tim50 (476 AAs), human (h) TIMM50 (353 AAs), and TbTim50 (423 AAs) (Figure 4A). They each possess a characteristic N-terminal MTS, although its length ranges from 22 to 44 AAs [58,67,69]. The MTS in fungal and human TIMM50 is cleaved from the precursor protein. The predicted size of TbTim50 is 47 kDa. Interestingly, the size of matured protein in mitochondria as well as the recombinant protein as found on a denaturing gel is around 50 kDa, suggesting that MTS cleavage is unlikely [58]. This finding warrants further investigation. The size of matured hTIMM50 is 40 kDa, which is smaller than that of Tim50 in fungi and trypanosomatids [69]. A longer isoform of TIMM50 (TIMM50a, with an additional 103 AAs at the N-terminus) also exists in human as a result of the translation of different transcripts from the same gene. The N-terminal extension in TIMM50a contains a nuclear localization signal; thus, Tim50a exists exclusively in the nucleus, particularly in the Cajal bodies [70]. Cajal bodies are sub-organelles in the nucleus where small nuclear RNA (snRNA) processing and modification occur [71]. snRNAs are generated in the nucleus, transported to the cytosol for further processing and assembly with Sm proteins to form snRNPs, and then transported back to the nucleus. In the nucleus, snRNPs enter Cajal bodies to undergo further processing and assembly with other proteins. After this final maturation step, snRNPs are released from the Cajal bodies into the splicing speckles in the nucleus. TIMM50a was found to associate with Cajal body proteins coilin, snRNPs, and SMN. Coilin competes with the Sm proteins, SMN, and snRNPs for Tim50a binding, suggesting that Tim50a helps release snRNPs from the Cajal bodies [70].

Tim50 possesses a single TMD near the N-terminal region. The TMD in hTim50 is within AA 66–88, while the TMD in ScTim50 and NcTim50 is within the AA 112–132 and 171–191, respectively. The smaller N-terminal domain is exposed to the matrix while the larger C-terminal domain is exposed to the IMS. Unlike Tim50 in other eukaryotes, TbTim50 possesses a weaker TMD (AA 285–310) (Figure 4A). Despite the unusual position and the weaker nature of this TMD, TbTim50 has been shown to be an integral membrane protein that is resistant to alkali treatment of isolated mitochondria [58]. In addition, a similar position of TMD has been predicted by TMpred server in Tim50 homologues in other trypanosomatids (Figure 3), suggesting that it could be a trait for Tim50 in this group.

We found that the membrane topology of TbTim50 is N-in and C-out (unpublished), which resembles that of Tim50 homologues in other eukaryotes. A major characteristic of Tim50 in all species is the presence of a C-terminal phosphatase-like motif resembling that in transcription factor TFIIF-stimulated CTD phosphatase. CTD phosphatase belongs to a special group of protein phosphatases that dephosphorylate S residues at the C-terminal domain of the large subunit of RNA polymerase II [72,73]. The catalytic site of these phosphatases contains the signature motif DXDX(T/V) where X represents any AA. However, since fungal Tim50 does not have a perfect motif, it does not possess phosphatase activity [35,36]. On the other hand, recombinant hTIMM50 possesses a dual-specific phosphatase activity [69]. Similarly, recombinant TbTim50 can hydrolyze both threonine and tyrosine phospho-peptides. Mutating D^242^ and D^244^ to A in the first DXDX(T/V) motif in TbTim50 abolished its phosphatase activity [58]. We recently discovered that in addition to phospho-peptides, recombinant TbTim50 also hydrolyzes phosphatidic acid to diacylglycerol and phosphate [74]. Mutating D^242^ and D^244^ to A also abolished the PA phosphatase (PAP) activity. TbTim50 also possesses a second ^345^DLDRV^349^ motif; however, mutation of the D^345^ and D^347^ to A only moderately reduced the TbTim50 phosphatase activity for both substrates. It has been shown that the first motif is also responsible for TbTim50 to bind PA and mutation in the second motif reduced the binding moderately (unpublished data). Therefore, the first motif is likely at the catalytic site of TbTim50 phosphatase and the second motif could be the auxiliary binding site for different substrates. In eukaryotes, PAP activity has been found in different lipin isoforms, which also possesses a similar DXDX((T/V) motif [75,76]. Lipin is an aspartate-based phosphatase that belongs to the haloacid dehalogenase (HAD) superfamily [75,76,77]. The HAD family of phosphatases or hydrolases have a wide spectrum of substrates that includes proteins, lipids, small molecules, and metabolites [77] (Table 2). Multiple sequence alignment and phylogenetic analysis revealed that trypanosomatid-Tim50 is grouped with Tim50s from other eukaryotes and is distinctly separated from other HAD-family proteins like lipin [75], small CTD-phosphatases (SCP1, FCP1) [78], ubiquitin-like domain containing CTD phosphatase (UBLCP) [79], and Dullard phosphatase [80] (Figure 3). Therefore, it seems TbTim50 is a special kind of HAD phosphatase with both protein- and lipid phosphatase activities and in addition plays role in protein translocation.

### 4.1. Tim50 tertiary Structure

The IMS-exposed region of ScTim50 consists of a protease-resistant core (AA 164–361) and a C-terminal presequence-binding domain (PBD) within AA 395–476 (Figure 4A). The folded PBD is in contact with the core domain that also interacts with the presequence [81,82]. Analysis of the crystal structure of the core domain revealed five α-helices (A1–A5) and nine β-strands (B1–B9). B1, B4, B5, B8, and B9 are at the center, whereas B2 and B3 with a short loop form a β-hairpin structure that protrudes from the surface of ScTim50 [81,82] (Figure 4B). This extended β-hairpin structure interacts with the N-terminal region of Tim23 that is exposed to the IMS. Near the β-hairpin structure, the IMS domain of Tim50 contains a large groove that recognizes the presequence during protein translocation. After emerging from the TOM complex, the presequence is recognized by the C-terminal PBD of Tim50. The preprotein is then transferred to the central groove of the Tim50 core domain to be handed over to the Tim23 channel. hTIMM50 does not have a C-terminal PBD; however, the β-strand protrusion and the nearby groove region are conserved [83] (Figure 4A,B). Fluorescence anisotropy, ANS-binding, and fluorescence quenching studies of purified hTIMM50_IMS_ revealed a compact structure in alkaline pH that became disordered in acidic pH, particularly when the pH went below 5.0 [83]. It is anticipated that the flexible structure of Tim50 in the acidic environment of the IMS is required for its dynamic conformational changes that happen during the transfer of preproteins from the TOM to the TIM23 complex.

Analysis of the predicted secondary structure of TbTim50 showed this protein to also possess multiple α-helix, β-sheet, and coiled-coil structures resembling those in yeast Tim50 (Figure 4C). Structural modeling of TbTim50 showed that the β-hairpin loop found in yeast Tim50 does not overlap well with that in TbTim50 (Figure 4C). However, the central core region formed by multiple β-sheets is very similar in both proteins. Furthermore, the groove region lateral to the β-hairpin loop is also conserved. The active site of many HAD phosphatases also possesses a similar substrate-binding groove with the signature sequence hhhhDxDx(T/V)(L/I)h (h, hydrophobic residue) [77]. The first D residue in this motif acts as a nucleophile that forms a phosphoaspartate intermediate during hydrolysis of the phosphate group from a protein or lipid. This structural similarity indicates that Tim50 evolved from a HAD-family protein and acquired the ability to bind presequences in mitochondrial preproteins. Some homologues of Tim50, like hTIMM50 and TbTim50, retain the phosphohydrolase activity, while others like fungal Tim50 do not.

**Figure 5:**
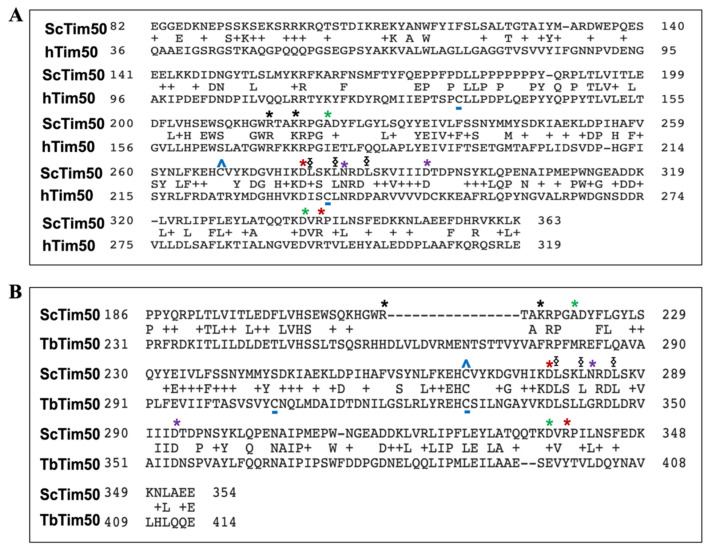
Primary sequence alignment of ScTim50 with hTim50 (**A**) and TbTim50 (**B**). Relatively conserved regions are shown. Identical AA residues are indicated. The conserved and non-conserved cysteine residues are indicated by blue ^ and underscore, respectively. Leucine residues within the conserved coiled-coil region (L279, L282, and L286 in ScTim50) are marked by ⧮. AA residues R214 and K217 located on the lateral side of the β-hairpin loop that are responsible for interaction with Tim23 are indicated by *. Three AA pairs that are important for the interaction between ScTim50 and ScTim23 are shown by asterisks of different colors (*, *, and *).

Another contrasting feature of fungal Tim50 is the number of C residues it possesses. In ScTim50, the sole C residue (C268) exists in the center of the presequence-binding groove [81,82]; however, it is not conserved in other Tim50 homologues. TbTim50 possesses six C residues (C22, C28, C108, C155, C306, and C329) spread along the entire protein. Among these, the last two Cs (C306 and C329) are located within the IMS-exposed region, while the rest are in the N-terminal region and in the TMD (Figure 5A,B). hTIMM50 has four Cs (C23, C133, C236, and C249), two of which (C236 and C249) are within the IMS-exposed region. The functional significance of these residues has not been determined in any system.

### 4.2. Role of Tim50 in Mitochondrial Protein Import

Functional studies in yeast have revealed ScTim50 as the receptor for the TIM23 translocase. The core domain of ScTim50 _IMS_ interacts not only with Tim23, but also with Tom22 and Tim21 [84,85,86]. Tom22_IMS_ and Tim21_IMS_ interact with Tim50 to connect the TOM and TIM23 complexes. Binding of Tim21 to Tom22 releases the precursor protein from the trans site of the TOM complex. Interaction between Tim50_IMS_ and Tim21_IMS_ depends on the presence of Tim23_IMS_. At the resting stage, these three proteins interact to close the TIM23 channel [84,85]. Recognition of the incoming presequence by Tim50_IMS_ dissociates Tim21. Interaction between ScTim50 and the N-terminal hydrophilic region of ScTim23 has been investigated. Crosslinking experiments revealed that AA residues Y70 and L71 in ScTim23_IMS_ interact with ScTim50_IMS_ [86]. Further analysis by alanine scanning mutagenesis revealed a stretch of five to six AAs in this region (AA 68–72) to be important for interaction with Tim50_IMS_, particularly E69 [84]. In case of Tim50, residues R214 and K217 (indicated by black * on the sequence in Figure 5A) on the lateral side of the β-hairpin loop are responsible for interaction with Tim23 [81]. The presequence-binding groove is adjacent to the β-hairpin structure. Therefore, binding of Tim50 to Tim23 facilitates the transfer of the presequence to Tim23_IMS_ for further translocation. Additional sites on Tim50_IMS_ responsible for its interaction with Tim23 have also been identified by mutagenesis analysis [86,87,88]. Tamura et al. showed L279, L282, and L286 located in one of the two coiled-coil regions in ScTim50 (indicated by ⧮ in Figure 5A,B) to be important for its interaction with ScTim23. Using random mutagenesis analysis, Dayan et al. identified three pairs of AAs—N283/D293, A221/D337, and D278/R339—located in two distinct patches on the surface of the Tim50 core as important for interaction with Tim23 (indicated by asterisks of the same color for each pair in Figure 5A,B) [88]. The mapping of these residues on the crystal structure model of the Tim50 core revealed that these two patches are close to residues R214 and K217 as well as to L279, L282, and L286. Thus, it appears that the flexible Tim23_IMS_ interacts with at least two regions in the Tim50 core domain. As such, mutating N283/D293, A221/D337, and D278/R339 may change the conformation of Tim50 and hamper its interaction with Tim23 [88]. The Tim50 core domain also interacts with the first TMD of Tim23 [89]. This interaction depends on cardiolipin levels in the MIM [90]. Malhotra and colleagues demonstrated that Tim50_IMS_ interacts with the MIM and that cardiolipin facilitates this interaction [90]. The presence of cardiolipin in the MIM also favors the association of ScTim50 with the Tim23 channel region; this association persists upon presequence binding during protein translocation. Conversely, the absence of cardiolipin in the MIM favors dissociation of Tim50_IMS_ from the MIM and the TMD of Tim23. Therefore, presequence binding under such condition dissociates Tim50 from Tim23. As such, cardiolipin plays a critical role in preprotein translocation. The N-terminus of Tim50 that is exposed to the matrix facilitates attachment of the PAM complex to the TIM23 complex by recruiting Pam17 [91]. Pam17 is a yeast-specific component of the TIM23 translocase (Figure 2A). It is an integral IM protein with two TMDs. Both the N- and C-termini are exposed to the matrix. Recruitment of Pam17 by activated TIM23 initiates the assembly of other motor components at the preprotein translocase to translocate matrix-targeted proteins. For translocation of MIM proteins containing the hydrophobic sorting sequence, Pam17 dissociates from TIM23 when the hydrophobic region of the precursor protein reaches the TIM23 channel. At this point, Mgr2 recruits Tim21 to the TIM23 translocase, and the channel opens laterally for translocation of the protein to the MIM [92].

The dynamics and action of Tim50 are less understood in human and in other systems. Sequence alignment revealed that AAs critical for the ScTim50-ScTim23 interaction are primarily conserved in hTIMM50 (Figure 5A), suggesting a similar mechanism of action for the human homologue. However, many of these residues are not conserved in TbTim50, particularly R214 and K217 (Figure 5B) located in the β-strand protrusion. Furthermore, there is a stretch of 17 AAs in this region that is unique to TbTim50 (Figure 5B). However, this divergence in TbTim50 is not unexpected because *T. brucei* lacks Tim23 but has TbTim17, which possesses a much shorter hydrophilic N-terminal region (30 AAs) than the 100-AA N-terminal region in ScTim23 [35,36]. Therefore, it is reasonable to postulate that the regions in TbTim50 that interact with TbTim17 are different. However, some other AAs like L279, L282, L286, D278, D285, and D293 within the coiled-coil region of ScTim50 are conserved in TbTim50 (Figure 5B), suggesting that this region may interact with other TbTims like TbTim17.

The role of TbTim50 in mitochondrial protein translocation remains largely unexplored. By overexpressing HA-tagged TbTim50 in *T. brucei*, we were able to co-immunoprecipitate TbTim17 and TbTim50-HA from the mitochondrial lysate [58]. Yeast two-hybrid analysis also showed TbTim17 to interact directly with TbTim50 (unpublished data). Functional studies showed TbTim50 to play a role in mitochondrial protein import [58]. In vitro protein import assay revealed *TbTim50* knockdown (KD) to inhibit the import of N-terminal signal-containing but not internal signal-containing nuclear-encoded mitochondrial proteins [58], indicating a similarity in substrate specificity among TbTim50, ScTim50, and hTim50. Furthermore, *TbTim50* KD reduced mitochondrial membrane potential in *T. brucei* while overexpression increased it [58,74,93]. Thus, like Tim50 in other eukaryotes, TbTim50 is required for maintaining the permeability barrier of the MIM in *T. brucei*. Therefore, additional information on the structure of TbTim50 and its interaction with TbTim17 is necessary to understand how TbTim50, a relatively conserved Tim, functions in a divergent translocase complex in *T. brucei*. One major obstacle in such studies is the need to tightly control TbTim50 expression. Both under- and overexpression results in a loss of cellular fitness and additional effects on cellular processes [93,94], which we will discuss next.

## 5. The Role of Tim50 in Other Cellular Processes

Multiple lines of evidence indicate that Tim50 is linked to various cellular functions (Table 3). Tim50 levels directly correlate with the growth and proliferation of various types of cancer cells as well as plant tissues [95,96]. Studies have shown that TIMM50 depletion in human cells to decrease mitochondrial membrane potential and accelerate apoptosis-mediated cytochrome C release [69]. On the other hand, TIMM50 overexpression increased mitochondrial membrane potential and induced apoptosis, the latter which co-expression of anti-apoptotic protein p35 could suppress [68]. In other words, both over- and under expression of TIMM50 can cause apoptotic cell death. Downregulation of TIMM50 by anti-sense RNA during early development in zebrafish embryos caused neurodegeneration, dysmorphic heart features, and reduced motility due to apoptosis [68]. In Drosophila, Tim50 mutation resulted in tiny flies, indicating a role for Tim50 in fly growth and development [69].

Mutation in TIMM50 is associated with several inherited health disorders in humans [104]. The unstructured region of TIMM50_IMS_ plays a critical role in steroidogenesis in gonadal tissues [98]. We recently found decreased TbTim50 levels to trigger signaling events that can alter the metabolic pattern [94] in the procyclic form (PF) of *T. brucei*. We also found TbTim50 to be associated with cell-cycle regulation and parasite infectivity in the bloodstream form (BF) [74]. The mechanism of action of Tim50 in these cellular functions is not well understood. The current school of thought is that Tim50 loss causes a reduction in mitochondrial membrane potential, which may disrupt many mitochondrial processes like protein import, respiration, and ATP production. These disruptions then lead to an accumulation of reactive oxidative species (ROS) in the mitochondria, which may trigger signaling events that are detrimental to the cells. More studies are necessary to investigate this avenue.

Recently, it has been shown that ScTim50 and hTIMM50 are substrates for mitochondrial phosphatase Pptc7 [108]. ScTim50 phosphorylation reduces its protein import capability. While phosphorylated TbTim50 also exists, the effect of this phosphorylation is unknown. Furthermore, there is no information thus far on any kinase that phosphorylates fungal, human, and *T. brucei* Tim50. As described above, hTIMM50 and TbTim50 possess phosphatase activity, but the role of this phosphatase remains elusive. Here, we discuss in detail some of the unique cellular functions associated with Tim50.

### 5.1. The Role of TIMM50 in Steroidogenesis

Tim50 plays a unique and essential role in steroidogenesis [98]. Unlike peptide hormones, steroid hormones are not stored in secretory vesicles to be released when the need arises [109]. Instead, steroids are synthesized at a high rate in a process known as acute steroidogenic response upon stimulation to meet demand [110]. The precursor of all steroid hormones is cholesterol that enters cells from blood via endocytosis mediated by the LDL-receptor and/or the scavenger receptor [109,110]. Cholesterol is also synthesized in cells de novo. Both cholesterol synthesis and uptake are tightly regulated by sterol-responsive element binding proteins (SREBPs) [111]. Once taken up from blood, cholesteryl esters in various lipid particles are hydrolyzed in the endo-lysosomal compartment to yield free cholesterol. Free cholesterol then enters the cytosol with the help of Niemann-Pick proteins 1 and 2 (NPC1 and NPC2) [112]. From the cytosol, cholesterol is transported to subcellular organelles like endoplasmic reticulum (ER) and mitochondria by vesicular fusion or by direct transfer via certain sterol-binding proteins. In mitochondria, the first step of steroidogenesis is the transfer of cholesterol from the MOM to MIM by steroidogenic acute regulatory protein (StAR) [113]. In the MIM, cholesterol is converted to pregnenolone by cholesterol side chain cleavage enzyme CyP450scc and further converted to progesterone by 3 β-hydroxysteroid dehydrogenase type 2 (3 β-HSD2). Pregnanolone also serves as the starting material for other steroid hormones like aldosterone, estradiol, and cortisone [98].

TIMM50 interacts with CyP450scc and 3 β-HSD2, two major steroidogenic enzymes in mitochondria [98,99,100]. Cyp450scc is a MIM protein with a cleavable N-terminal targeting signal that is processed twice. The C-terminus of the mature protein remains embedded in the TIM23 complex, whereas the flexible N-terminal domain is exposed to the IMS. The conformation of this N-terminal domain changes dynamically to accommodate cholesterol binding and release. Tim50_IMS_ is involved in the interaction with the flexible domain of Cyp450ssc to help change the conformation of the latter. Recent studies revealed that AA residues 164–168 of TIMM50 interact with AA residues 141–146 of CyP450ssc [98]. Mutations in these regions have been shown to cause a total loss of CyP450ssc metabolic activity and steroidogenesis. Unlike CyP450ssc, 3 β-HSD2 is an IMS protein peripherally associated with the IM. 3 β-HSD2 has an uncleavable N-terminal targeting signal that promotes mitochondrial entry and targeting to the TIM23 complex. However, this protein does not reach the MIM or the matrix. Instead, it is released into the IMS [99]. Both a dehydrogenase and an isomerase, 3 β-HSD2 helps convert pregnanolone to progesterone [100]. Due to these capabilities, the conformation of 3 β-HSD2 is interchangeable between the folded and unfolded conformation. It has been shown that unfolded 3 β-HSD2 needs to interact with TIMM50 for stability. TIMM50 depletion has been shown to cause rapid 3 β-HSD2 degradation and hamper steroidogenesis. Therefore, TIMM50_IMS_ acts as a chaperone by interacting with partially unfolded 3 β-HSD2 and CyP450ssc, thus playing a crucial role in two early steps of steroid hormone production.

### 5.2. Tim50 Mutations in Genetic Disorders

In the last five years, there have been case reports indicating that childhood epileptic encephalopathy, developmental retardation, and 3-methylglutaconic aciduria (3MGA-uria) are linked to inherited mutations in the *TIMM50* gene [104,105,106]. Shahrour et al. reported cases of two pairs of siblings from two unrelated families, who presented with severe intellectual disability, seizure disorder, and 3MGA, accompanied by elevated lactate level. Whole exome sequencing (WES) identified two homozygous mutations in *TIMM50*, R217W and T252M (numbered according to AA positions in the long open-reading frame of *TIMM50*) [105]. Both residues are evolutionarily conserved and located in Tim50_IMS_. To examine the effect of these mutations on Tim50 function, the authors created a homologous mutation in ScTim50 (R159W), which corresponds to R217W in TIMM50 [105]. Interestingly, this mutation did not have any effect on cell growth and mitochondrial protein import in yeast. Since wildtype hTim50 could not restore the viability of a Tim50-deficient yeast mutant, these negative results could not rule out the importance of R217 and T252 in TIMM50 function. Additionally, Mir et al. found a homozygous mutation (T252M) in Tim50 in a pediatric patient with similar symptoms [106]. 3MGA is a hallmark of mitochondrial defect, specifically of mitochondrial membrane dysfunction [114]. Patients with mutations in tafazzin (TAZ), the enzyme that modulates levels of mitochondrial membrane-specific lipid cardiolipin, also show symptoms like ataxia, cardiomyopathy, and 3MGA [115]. These findings indicate a connection between TIMM50 and cardiolipin composition.

Reyes and colleagues [104] reported another case of disease-causing TIMM50 mutation. In this study, they used WES to identify compound heterozygous pathogenic mutations in *TIMM50* in a pediatric patient suffering from rapidly progressing severe encephalopathy as well as elevated levels of lactate in plasma and cerebrospinal fluids. The patient died of cardiorespiratory arrest at the age of 32 months. The mutations identified were (335C>A) S112* (an N-terminal truncation mutation) and (569G>C) G190A (in the TMD). The authors observed a marked reduction in TIMM50 levels in the patient’s fibroblasts with concomitant reduction in levels of other components of the TIM23 complex, such as TIMM17A, TIMM17B, TIMM23, and DNAJC19. Additionally, mitochondrial membrane potential was reduced moderately and levels of ROS increased [104]. Consequently, superoxide dismutase 2 (SOD2) and aconitase 2 (ACO2) levels increased. Import of nuclear-encoded mitochondrial protein TFAM was reduced but import of AAC1 was not, indicating a reduction in only the import of TIMM50 substrates. In addition, these mutations also resulted in a lower respiration rate, particularly that of respiration coupled with ATP production. VDAC1 and LC3 levels were upregulated with concomitant reduction in p62 levels, indicating an enhanced autophagic response. Furthermore, expression of wildtype TIMM50 in the patient’s fibroblast cells reversed most of these defects, indicating that these symptoms are related to disruption of TIMM50 function. Mutations in other mitochondrial genes like *RNASEH1* and iron-sulfur cluster subunit U (*ISCU*) did not cause these defects even though mitochondrial membrane potential was reduced due to the depletion of these proteins [104]. This study showed that these defects are not solely due to reduced mitochondrial membrane potential, and that the role of TIMM50 in mitochondrial function is more complex than it is currently known.

Furthermore, Tort et al. reported a case of a 17-year-old boy who had suffered from epileptic syndrome and abnormal eye movement in his childhood and received anti-epileptic treatment [107]. However, relapses of neurological regression along with scoliosis and osteoarticular problems rendered him wheelchair bound. Biochemical investigation showed a persistent increase in 3-MGA levels in his urine. WES analysis identified mutations R114Q and G269S in Tim50_IMS_. These mutations caused a decrease in TIMM50 levels [107] as well as defects in mitochondrial morphology and mitochondrial network architecture. In addition, the authors also observed a defect in the assembly of oxidative phosphorylation supercomplexes, which could be linked to alteration in mitochondrial membrane lipid composition.

### 5.3. TIMM50 in Cardiac Function

Pathological cardiac hypertrophy and dilated cardiomyopathy (DCM) leading to heart failure are due to the dysfunction of various intracellular mechanisms [116]. Among these, inadequate response to oxidative stress is one of the main causes for cardiac hypertrophy. Mitochondria are the major source of ROS. Mitochondrial dysfunction in aging and certain pathological conditions leads to ion leakage from oxidative phosphorylation, resulting in increased ROS levels [117]. Tang et al. discovered TIMM50 as a novel repressor of pathological cardiac hypertrophy [101]. The authors observed a downregulation of TIMM50 expression in a murine model of DCM as well as in cardiomyocytes with angiotensin II-induced hypertrophy. TIMM50 overexpression attenuated these effects. Furthermore, *TIMM50*-knockout (KO) mice were prone to cardiac hypertrophy induced by aortic band surgery, while heart-specific *TIMM50* transgenic mice were protected. The authors also observed an increase in ROS levels and reduced activity of the respiratory complexes in mitochondria of heart tissues from *TIMM50*-KO mice, which rendered these animals more susceptible to cardiac hypertrophy. Treating these animals with antioxidant N-acetyl cysteine (NAC) ameliorated this diseased phenotype [101]. Treatment with NAC also attenuated the phosphorylation of apoptosis signal-regulating kinase 1 (ASK1) and downstream activation of the MAP kinase pathway caused by *TIMM50* KO. These findings indicate that Tim50 loss induces apoptosis, which is detrimental to heart function. These findings also indicate that TIMM50 protects heart function. However, how mitochondrial dysfunction due to TIMM50 loss affects cellular signaling is not well understood. Furthermore, the relationship between TIMM50 expression and cardiac hypertrophy warrants further investigation.

Cardiac function also depends on mitochondrial membrane phospholipids. The mitochondrial membrane is characterized by the presence of cardiolipin. Cardiolipin contains two phosphatidyl glycerides joined by a glycerol moiety; thus, it has four fatty acid chains. Varying fatty acid compositions give rise to diverse species of cardiolipin [118] in different tissues. The heart mitochondria contain a definite pool of cardiolipin with linoleic acid for all four acyl chains. Alteration in the amount and type of cardiolipin is linked to cardiac dysfunction and heart failure. cardiolipin is critical for many mitochondrial functions, including the assembly of respirasome supercomplexes, assembly of mitochondrial carrier proteins, mitochondrial fission and fusion, mitophagy, and metabolic regulation [119]. Mitochondrial protein translocases also require cardiolipin for their assembly and function. ScTim50_IMS_ interacts with cardiolipin in the MIM to prevent proton leakage through the TIM23 channel [90]. Cardiolipin is synthesized in the IM via multiple enzymatic steps, starting from phosphatidic acid (PA) translocated from the ER through ER-mitochondrial junction sites (ERMES). After initial synthesis, cardiolipin is remodeled by several deacylation/reacylation steps for maturation. Mutations in cardiolipin-remodeling enzyme TAZ causes Barth syndrome, a serious genetic disorder [119,120]. Patients with Barth syndrome present with DCM, loss of mitochondrial membrane potential, decrease in respiratory chain supercomplexes, growth retardation, and higher 3-MGA levels in their urine [119,120]. This phenomenon shows that cardiolipin is critical for heart function. Furthermore, since TIMM50 depletion also showed similar phenotypes, it suggests that Tim50 plays role in cardiolipin composition in mitochondria.

Another genetic disorder associated with mutation in DNAJC19, a component of the PAM complex, is also linked to alteration in cardiolipin composition and abnormal mitochondrial function. Sengers syndrome, an autosomal-recessive disorder manifested as hypertrophic cardiomyopathy, is due to mutation in acyl glycerol kinase (AGK) [121,122]. AGK is a mitochondrial lipid kinase expressed at high levels in the heart. AGK phosphorylates both mono- and diacyl glycerol to form lysoPA and PA, respectively, both precursors for cardiolipin synthesis [116]. Thus, mutation in AGK reduces cardiolipin levels and affects mitochondrial function. These defects resemble those in Barth syndrome. AGK is also a component of the human TIM22 complex required for importing mitochondrial carrier proteins. Thus, AGK mutation also reduces levels of the mitochondrial ATP/ADP carrier, which is critical for mitochondrial ATP production [123]. Overall, heart diseases are intimately linked to cardiolipin levels and mitochondrial function. Heart mitochondrial dysfunction due to Tim50 loss further indicates a connection between Tim50 and cardiolipin levels. It is possible that cardiolipin loss reduces TIMM50 stability or vice versa. Investigation in this aspect is ongoing.

### 5.4. TIMM50 in Cancer

Sankala and colleagues were the first to demonstrate an association between TIMM50 and cancer [102]. P53 is a well-known tumor suppressor protein that controls cell-cycle progression, DNA integrity, and cell survival. Certain mutations in P53 not only abolish its tumor-suppressive function, but also attribute additional functions to the protein as well as promote oncogenesis and chemoresistance [102,124]. Sankala et al. observed an upregulation in TIMM50 levels in mitochondria in lung cancer cell line H1299 as well as in breast cancer cell lines MDA-MB-468 and SK-BR-3, all with P53 gain-of-function mutations (R175H and R273H). Conversely, breast cancer cell line MCF-10, which expresses wildtype P53, did not show this upregulation. These P53 mutants increased *TIMM50* promoter activity by enhancing histone acetylation as well as interaction between the promoter and transcription factors Eis-1, CREB, and CBP. shRNA-induced reduction of TIMM50 levels reduced growth and chemoresistance of the P3 gain-of-function mutant cancer cells but not cancer cells that lack P53. It is possible that an increase in TIMM50 levels enhances mitochondrial import of proteins that increase mitochondrial activity, thus promoting cell growth. Furthermore, TIMM50 maintains mitochondrial permeability barrier and inhibits apoptosis, characteristics that may confer chemoresistance to cancer cells [102]. Subsequent studies demonstrated upregulation of TIMM50 levels as a survival strategy for various types of cancer cells.

In a 2016 publication, Gao et al. reported an elevation in TIMM50 levels in breast cancer cell lines and in patient-derived tissue samples. Suppression of TIMM50 by shRNA reduced cell proliferation and increased apoptosis by decreasing mitochondrial membrane potential [103]. Similarly, Zhang et al. showed in non-small cell lung carcinoma (NSCLC) that TIMM50 levels directly correlated with large tumor size, advanced TNM stage, lymph node metastasis, and poor overall survival [95]. The same study also showed upregulation of TIMM50 levels in NSCLC to be associated with upregulation of cyclinD1 levels [95]. Cyclin D1 promotes G1 progression in the cell cycle. TIMM50 upregulation also increased the level of Snail, a repressor for cell-cell junction protein E-cadherin. Consequently, E-cadherin levels decreased, and cell migration was enhanced. Changes in TIMM50 levels in the mitochondria of these cells triggered signaling events mediated by ERK and P90RSK phosphorylation. Inhibition of ERK activity counteracted the upregulation of cyclin D1 and Snail in TIMM50-overexpressing cells, suggesting a role for TIMM50 in integrating mitochondrial function with signaling events that regulate cell growth and proliferation [95]. However, how this occurs is unclear. Recent findings indicate that microRNA miR-7 regulates *TIMM50* expression [125]. miR-7 is a tumor-suppressive microRNA that targets several genes that facilitate cell growth and inhibit apoptosis, such as *KLF4* and *BCL2*, among others. However, the connection between miR-7 and *TIMM50* expression only became known when the latter was revealed as a Mir-7 target. Yang et al. demonstrated the antitumor effect of miR-7 in rhabdomyosercoma, a pediatric soft tissue cancer. Inhibition of apoptosis and necroptosis reduced miR-7-induced cell death. Using TargetScan to predict miR-7 targets, the authors identified three mitochondria-related genes: *SLC25A37* (encodes a mitochondrial iron transporter), *VDAC1*, and *TIMM50*. They further confirmed the downregulation of target transcripts and proteins upon miR-7 treatment. Using luciferase reporter assay, the authors confirmed the 3′-UTR of the *SLC25A37* and *TIMM50* transcripts as direct targets of miR-7 [125]. Other studies revealed circular RNA ciRS-7 and long non-coding RNA SOX21-AS1 as sponge RNAs that reduce miR-7 levels in different cancer tissues [126]. Taken together, findings from these studies indicate that cancer cells produce more TIMM50 than normal cells, either by increasing the *TIMM50* promoter activity or by inhibiting the degradation of the *TIMM50* transcript, to increase mitochondrial activity. These enhancements are integrated with certain cellular signaling events to facilitate cell growth and metastasis.

### 5.5. The Role of TbTim50 in T. brucei Biology

During its digenetic life cycle, *T. brucei* undergoes various differentiation steps to adapt to the diverse environments in two different hosts [127]. In the fly vector, the parasite lives in the midgut as the procyclic form (PF) and travels to the salivary glands where it transforms into the infective metacyclic form [127]. In a mammalian host, *T. brucei* multiplies extracellularly in the bloodstream and tissues as the bloodstream form (BF). At the peak of each wave of parasitemia, the dividing long-slender (LS) BF transforms into the non-dividing stumpy (ST) BF via a density-sensing phenomenon [128]. This non-dividing ST form ultimately perishes, and a new wave of the LS form appears with antigenically different surface proteins to evade the host’s immune defense. The ST form is the pre-adaptive form that quickly and synchronously transforms into the PF upon transfer to the fly gut through fly bites. The PF utilizes various carbon sources (carbohydrates and amino acids) to produce energy, primarily via oxidative phosphorylation [129,130]. On the other hand, the BF depends solely on glucose as its energy source and suppresses activities in mitochondria like oxidative phosphorylation and Krebs cycle [129,130]. Instead, respiration in the BF occurs via cytochrome-independent trypanosome alternative oxidase (TAO) in mitochondria and is not coupled with ATP production [129,130]. As such, the BF hydrolyzes ATP by reversing the action of ATP synthase to generate mitochondrial membrane potential [131,132].

Compared to alteration in the levels of other TbTims in the TbTIM complex, changes in TbTim50 levels have a broader impact on cellular functions in *T. brucei* [58,74,94]. In the PF, *TbTim50* KD moderately reduces cell growth in normal glucose concentration. When the cells are deprived of glucose, the reduction in cell division is more pronounced since the cells rely more heavily on mitochondrial activity for survival [94]. On the other hand, growth inhibition is more pronounced in the BF upon *TbTim50* KD even in normal glucose concentration since this form depends solely on glycolysis for growth and survival [74]. These findings indicate that TbTim50 could somehow inhibits cell growth of BF by different mechanisms than PF. On the other hand, TbTim50 is required to maintain mitochondrial membrane potential in both the PF and BF [74,94]. In the PF, TbTim50 depletion reduces ATP production by oxidative phosphorylation, while substrate-level phosphorylation increases [94]. Nevertheless, overall ATP levels decrease, and AMP levels increase. Consequently, AMPK is activated by phosphorylation, which is associated with possible alterations in metabolic pattern as supported by global proteomics analysis [94]. Interestingly, *TbTim50* KD increases the abundance of another HAD-family phosphatase, phosphotyrosyl phosphatase-interacting protein (PIP39) in the PF [94]. PIP39 is a developmentally regulated protein in *T. brucei*. Its expression level is low in the replicating SL BF but high in the non-dividing ST BF and in the PF [128,133]. PIP39 is a substrate for phosphotyrosyl phosphatase (PTP). In the BF, both PTP and PIP39 are localized near the flagellar pocket [133] formed by invagination of the plasma membrane near the flagellar basal body, the only site for both endo- and exocytosis in *T. brucei*. However, in the PF, PTP is inactivated, causing PIP39 to be phosphorylated and transported to glycosomes [128]. Glycosomes are peroxisome-like organelles that encapsulated most of the glycolytic enzyme in trypanosomatids [134]. The role of PIP39 is less understood in the PF. It is puzzling to observe that PIP39 expression is inversely related to TbTim50 levels in mitochondria. How these two phosphatases are connected is unclear. We previously reported a likely connection between PIP39 expression and cellular energy status and/or redox balance [94]. Specifically, upregulation of PIP39 due to *TbTim50* KD enabled PF cells to tolerate external oxidative stress better than wildtype cells, while knocking down both *TbTim50* and *PIP39* reduced this tolerance to wildtype level [94]. Therefore, these two similar phosphatases localized in two distinct subcellular regions are involved in maintaining cellular homeostasis. However, the mechanism remains to be elucidated.

The BF produces ATP via glycolysis and hydrolyzes ATP in mitochondria and consequently pump protons from the matrix to the IMS [131,132]. In addition, mitochondrial ADP/ATP carrier protein AAC/MCP5 imports ATP (-4) and exports ADP (-3), thus also plays role in maintaining the electrogenic potential of the MIM [131,132,135]. However, since AAC is not a substrate of TbTim50, *TbTim50* KD did not change AAC levels [58,94]. In addition, ATPase subunits were also not reduced significantly due to TbTim50 RNAi [94]. Nonetheless, we observed TbTim50 depletion to reduce mitochondrial membrane potential and cause an energy crisis in the BF, as a consequence AMPK phosphorylation was increased [74]. Therefore, the question raised how TbTim50 is involved to maintain mitochondrial membrane potential in BF. Loss of mitochondrial membrane potential due to TbTim50 depletion is a phenomenon common to all eukaryotes studied so far [58,68,69]. In yeast, TbTim50_IMS_ binds to the hydrophilic IMS-exposed N-terminal region of Tim23 as well as to the first TMD of Tim23 in the presence of cardiolipin in the MIM [90]. This interaction keeps the channel closed in the absence of preproteins and maintains the permeability barrier of the MIM [97]. However, if this is true for Tim50 in other eukaryotes is not known. Particularly, in *T. brucei*, it is not clear how TbTim50 interacts with TbTim17, the counterpart of Tim23 in yeast.

Recent studies showed that besides the protein phosphatase activity, TbTim50 can bind to and hydrolyzes PA, thus possesses PA phosphatase activity. Moreover, TbTim50 loss in the BF reduces mitochondrial cardiolipin levels [74]. Therefore, TbTim50 is speculated to play a role in either cardiolipin synthesis or maintaining its stability. Since cardiolipin is essential for MIM integrity and the assembly/stability of various mitochondrial membrane protein complexes, it is conceivable that cardiolipin loss due to *TbTim50* KD reduces mitochondrial membrane potential in *T. brucei*. It has been shown recently that although the BF does not depend on mitochondrial ATP production, cardiolipin loss is detrimental to its cellular ATP production [136]. This is because electron transfer via TAO in mitochondria is essential for aerobic glycolysis in the BF. Loss of MIM integrity due to reduction of cardiolipin levels reduces TAO levels and disrupts electron transfer. This phenomenon could explain the energy crisis in the BF due to *TbTim50* KD.

TbTim50 depletion also arrests the BF cells in the G1 phase and decreases the population in the G2/M phase of the cell cycle [74]. Therefore, TbTim50-depleted *T. brucei* failed to establish infection in mice and rats [74]. *TbTim50* KD changed the morphology of the LS monomorphic BF. In addition, multiple ST-specific transcripts were also upregulated under such condition. Taken together, these findings indicate that TbTim50 function either directly or indirectly could be linked to *T. brucei* differentiation. Since AMPK activation can trigger SL-to-ST transition, it is likely that increased AMPK phosphorylation due to *TbTim50* KD triggers this transition. Therefore, the senescent BF cells due to *TbTim50* KD causes their rapid clearance from the host, subsequently reducing parasite infectivity. Therefore, TbTim50 is a potentially important target for treating African trypanosomiasis.

While TbTim50 is essential for mitochondrial function in both the PF and BF, TbTim50 depletion results in different phenotypes in these two forms. The PF is more metabolically flexible; thus, it can compensate for the decrease in its mitochondrial function by altering its metabolic pattern. On the other hand, the BF is less flexible, and its mitochondrial fitness is intimately linked to overall cellular fitness. Therefore, it is important to elucidate how TbTim50 impacts broader cellular function in *T. brucei*.

## 6. Conclusions

Tim50 is a conserved component of the preprotein trannslocase in mitochondria. The unique C-terminal domain of Tim50 possesses a typical Rosmann fold structure similar to that found in the HAD phosphatase superfamily. The yeast Tim50 does not have the conserved motif and loses the catalytic activity, however, the motif is preserved in Tim50s in other eukaryotes like human and *T. brucei*. This C-terminal domain of Tim50 that is exposed in IMS performs various functions, like (1) acts as the receptor for preproteins, (2) behaves like a chaperone to bind certain unfolded proteins and protect these proteins from degradation, (3) interacts with MIM in cardiolipin-dependent manner, and (4) maintain mitochondrial membrane potential. Loss of Tim50 functions causes various disease pathologies and its overexpression favors cancer cell metastasis. Further investigation on Tim50 in different organisms will increase our understanding regarding how mitochondrial functions is integrated to various essential cellular processes in eukaryotes.

## Figures and Tables

**Table 1:** Components of the Mitochondrial protein import complexes in different organisms.

Complex	Yeast/Fungi	Human	Plant	Trypanosomatids
SAM/TOB	Sam50/Tob55, Sam35/Tob38, Sam37/Mas37, Mdm10	Sam50/Tob55, Metaxin 1, Metaxin 2	Sam50/Tob55, Sam37(Metaxin), Mdm10	Sam50/Tob55
TOM	Tom40, Tom22, Tom5, Tom6, Tom7, Tom20, Tom70	Tom40, Tom22, Tom5, Tom6, Tom7, Tom20, Tom70	Tom40, Tom9(Tom22), Tom5, Tom6, Tom7, Tom20, OM64 ^e^	Atom40 ^f^, Atom46 ^f^, Atom69 ^f^ Atom19 ^g^, Atom14 ^g^, Atom11 ^g^, Atom12 ^g^,
MIM	Mim1, Mim2			Atom36 ^h^
MIA	Mia40, Erv1		Mia40, Erv1	Erv1
Small Tims	Tim9, Tim10, Tim8, Tim13, Tim12	Tim9, Tim10a, Tim10b, Tim8, Tim13	Tim9, Tim10, Tim8, Tim13	TbTim9, Tbim10, TbTim11 ^g^, TbTim12 ^g^, TbTim13 ^g^, TbTim8/13 ^g^
TIM23	Tim23, Tim17, Tim50, Tim21	TIMM23, TIMM17, TIMM50	Tim23, Tim17, Tim50, Tim21	TbTim17, TbTim62 ^g^, TbTim54 ^g^, TbTim42 ^g^, TbTim50, ACAD ^g^, Rhomboid I ^g^, Rhomboid II ^g^, small TbTims ^h^
PAM	Pam18, Pam16, Pam17, Tim44, Hsp70, Mge-1	DnaJC19 ^a^, DnaJC15 ^a^, Magma ^b^, Mortalin/HSPA9 ^c^	Pam18, Pam16, Tim14, Tim44, Hsp70, Mge1	TbPam27 ^g^, Hsp70
TIM22	Tim22, Tim54, Tim18, Sdh3, Tim12	TIMM22, TIMM29 ^d^, AGK ^d^	Tim22	
OXA	Oxa1	Oxa1	Oxa1	Oxa1

^a^ Human DnaJC19 andDnaJC15 are functional homologues of yeast Pam18. ^b^ Magma is the homologue of yeast Pam16. ^c^ Mortalin/HspA9 is the mitochondrial Hsp70. ^d^ TIMM29 and AGK are the human specific components of the TIM22 complex. ^e^ OM64 is a plant specific receptor/translocase of the TOM. ^f^ Atom40 is an archaic homologue of Tom40 in *T. brucei*, Atom46 and Atom69 are functional homologues of Tom20 and Tom70, respectively. ^g^ Atom19, Atom14, Atom11, Atom12 and TbTim62, TbTim42, TbTim54, ACAD, RhomboidI, RhomboidII, are trypanosome-specific components of the ATOM and TbTIM complexes, respectively. TbPam27 is trypanosome specific. ^h^ Atom36 and small TbTims are orthologues of Mim1/Mim2 and small Tims, respectively. In *T. brucei*, small TbTims are part of the TbTIM17 complex.

**Table 2:** The HAD family of phosphatases.

HAD Family	Examples	Substrate	Function
RNA polymerase	^1^ FCP, ^2^ SCP1-3	*p*-Serine	Gene expression
C-terminal domain	Dullard	in proteins	regulation
Phosphatase	^3^ UBLCP1		
Polynucleotide	PNKP	3′-Phosphorylated	DNA repair
Kinase phosphatase		DNA termini	
Epoxide hydrolase	sEH2	Dihydroxylipid	Lipid metabolism
		phosphate, iso-	cholesterol
		prenoid phosphate	biosynthesis
Intracellular	^4^ cN-I-III	AMP, GMP	Nucleotide/
5′-Nucleotidase	^5^ cdN, ^6^ mdN	IMP	nucleoside balance
Phosphoserine	PSPH	L-3-Phosphoserine	Serine biosynthesis
Phospho hydrolase			
Eyes absent	EYA1-4	*p*-Tyrosine	Organ development
		in proteins	DNA repair
			Cell proliferation
HAD-like	pseudouridine 5′	Pseudouridine-P	Pseudouridine
Hydrolase	monophosphatase,	secretion	
	pyridoxal 5′-phosphate	Pyridoxal-P	Vitamin B_6_
	phosphatase	metabolism	
Lipins	Lipin1-3, ^7^ PAP	Phosphatidic acid	Lipid metabolism

^1^ fungal C-terminal domain (CTD) phosphatase, ^2^ small CTD-like phosphatase, ^3^ ubiquitine-like domain containing CTD phosphatase, ^4^ cytosolic 5′-nucleotidase, ^5^ cytosolic 5′-deoxynucleotidase, ^6^ mitochondrial 5′-nucleotidase, ^7^ phosphatidic acid phosphatase.

**Table 3:** Tim50 functions.

Organism	Functions	References
Yeast/Fungi	Presequence receptor of the TIM23 complex	[35,36,65,66]
	Maintain mitochondrial membrane potential	[84,85,97]
	Cardiolipin-dependent membrane association	[90]
Human	Presequence receptor of the TIM23 complex	[15,83]
	Maintain mitochondrial membrane potential	[68,69]
	Possesses protein phosphatase activity	[69]
	Promotes steroidogenesis	[98,99,100]
	Cardiac cell protection	[101]
	Cancer metastasis	[95,102,103]
	Genetic disorder	[104,105,106,107]
Zebra fish	Subunit of the TIM23 complex	[69]
	Maintain mitochondrial membrane potential	[69]
	Developmental regulation	[69]
Drosophila	Subunit of the TIM23 complex	[68]
	Maintain mitochondrial membrane potential	[68]
	Developmental regulation	[68]
Plant	Subunit of the TIM23 complex	[16,96]
	Regulation AMPK by phosphorylation	[96]
Trypanosoma	Required for mitochondrial protein import	[58]
	Maintain mitochondrial membrane potential	[58,93,94]
	Possesses protein phosphatase and Phosphatidic acid phosphatase activities	[58,74]
	ROS production	[93,94]
	Stress tolerance	[93,94]
	Cardiolipin levels	[74]
	Cell growth	[74,93,94]
	Cell cycle regulation	[74]
	Infection	[74]

## Data Availability

Not applicable.
