# Correction: Chaudhuri et al. Diverse Functions of Tim50, a Component of the Mitochondrial Inner Membrane Protein Translocase. *Int. J. Mol. Sci.* 2021, *22*, 7779

**DOI:** 10.3390/ijms23147496

**Published:** 2022-07-06

**Authors:** Minu Chaudhuri, Anuj Tripathi, Fidel Soto Gonzalez

**Affiliations:** Department of Microbiology, Immunology, and Physiology, Meharry Medical College, Nashville, TN 37208, USA; atripathi@mmc.edu (A.T.); Fgonzalez18@email.mmc.edu (F.S.G.)

The authors wish to make the following correction to this paper [1]:

Changes in Figure 5, because Figure 5B misprinted as a duplication of Figure 5A by authors mistakenly during the proofreading. The correct Figure 5 is shown below. Figure changes will not affect the description and conclusion of the manuscript.

The authors would like to apologize for any inconvenience caused to the readers by these changes. This correction was approved by the Academic Editor. The original publication has also been updated.

## Figures and Tables

**Figure 5:**
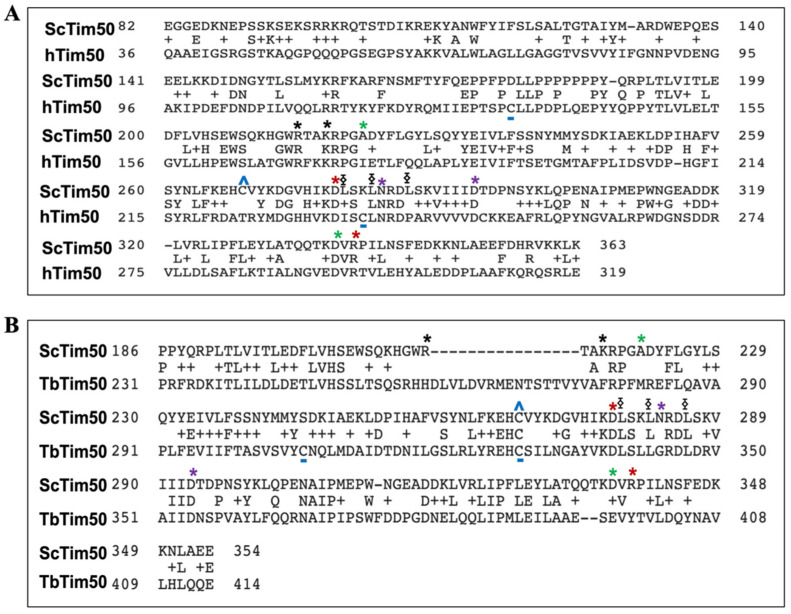
Primary sequence alignment of ScTim50 with hTim50 (**A**) and TbTim50 (**B**). Relatively conserved regions are shown. Identical AA residues are indicated. The conserved and non-conserved cysteine residues are indicated by blue ^ and underscore, respectively. Leucine residues within the conserved coiled-coil region (L279, L282, and L286 in ScTim50) are marked by ⧮. AA residues R214 and K217 located on the lateral side of the β-hairpin loop that are responsible for interaction with Tim23 are indicated by *. Three AA pairs that are important for the interaction between ScTim50 and ScTim23 are shown by asterisks of different colors (*, *, and *).
